# Text Messaging After HIV and Sexually Transmitted Infection Screening: Do Patients' Profiles Matter?

**DOI:** 10.1097/OLQ.0000000000000941

**Published:** 2018-11-08

**Authors:** Pénélope Troude, Christophe Segouin, Christelle Duteil, Marc Shelly, Elise de La Rochebrochard

**Affiliations:** From the * Service de Santé Publique, HU Saint-Louis—Lariboisière—Fernand Widal, AP-HP;; † Institut National d'Etudes Démographiques;; ‡ CeGIDD, HU Saint-Louis—Lariboisière—Fernand Widal, AP-HP, Paris; and; § Université Paris-Saclay, Univ. Paris-Sud, UVSQ, CESP, INSERM, Villejuif, Le Kremlin-Bicêtre, France

## Abstract

**Background:**

Text messaging after sexually transmitted infection (STI)/HIV screening may be a cost-effective means of improving patient care, but it may not be appropriate for all patients. This study aimed to explore the profiles of patients who did not participate in a short message service (SMS) program after STI/HIV testing.

**Methods:**

In October 2016, 396 patients in Paris were screened for STI/HIV and were invited to complete an anonymous self-administered questionnaire. Patients were offered the possibility of being notified by SMS after testing, 68% accepted (SMS group) and 32% did not (no-SMS group). Each of the 100 patients from the no-SMS group who had completed the questionnaire was matched with the next patient from the SMS group. Factors associated with nonparticipation in the SMS program were studied using conditional logistic regression models.

**Results:**

Participation in the SMS program was not related to STI screening characteristics (screening results and seriousness of the diseases screened) but seemed to be related to patient characteristics. In multivariate analysis, compared with patients in the SMS group, those in the no-SMS group were more often older, socially less favored (born in Africa or Asia, no university diploma, living outside Paris). They also more often declined to answer sexual questions, which could reflect a need for privacy and discretion.

**Conclusions:**

Although SMS after STI/HIV screening is well accepted, it does not suit all patients. Several contact options should be proposed to comply with patients' preferences and to reduce the risk of nondelivery of STI screening results.

## Abstract 2

Participation in a short message service program after sexually transmitted infection/HIV screening seemed quite good but varied according to patient profile. Several options should be proposed for delivering the results of sexually transmitted infection screening.

Text messaging is increasingly used to facilitate communication with patients in health care.^1,2^ It can be a tool for behavior change in disease prevention and management of chronic diseases.^1^ Interventions using mobile text messaging in health care have been shown to be effective in improving diabetes self-management, weight loss, physical activity, smoking cessation and medical adherence to antiretroviral therapy.^2^ Text messaging can also be used for delivery of medical test results,^1^ especially for HIV or other sexually transmitted infections (STIs).^3^ Notification by short message service (SMS) after STI screening may be a cost-effective means of improving patient care for STIs.^4–9^ Firstly, the texting notification protocol reduces delay between screening and treatment and decreases the proportion of untreated patients.^6,7,10^ For example, a study conducted in an inner London sexual health clinic found that texting decreased the time to treatment for genital *Chlamydia trachomatis* infection from 15.0 to 8.5 days.^6^ Secondly, as negative results are delivered by SMS, the texting protocol reduces the time medical staff spend on delivering negative results, freeing them to spend more time with patients with positive results. In a London clinic receiving 800 to 900 patients per month, it was estimated that the medical staff saved 46 hours a month by using text messaging notification.^6^

Text messaging after STI/HIV testing is now recommended in European guidelines.^11^ Texted information should use simple vocabulary to minimize risk of misunderstanding and should be short and discreet to be accepted by patients. When results are negative, patients can be informed by a message such as “*Your results are fine”* or “*All good”*^9,10^ and they do not need to return to the center. Such a feedback protocol ensures that all patients with negative results are informed of their screening results. This is not the case when the patient has to come back to the center, as 7 to 22% of patients fail to return after STI screening.^12–15^ When patients have positive results, the SMS invites them to come back to the center with texts such as “*Results now back”* or “*Hi (client's name) I need 2c U. Can U contact me? Thanx (clinician's first name)*”. Despite benefits to patient and medical staff, and agreement with guidelines for European countries, communication technologies are still perceived as under-used by health professionals for delivering HIV test results across European regions.^16^

The few studies that have explored patients' preferences showed an overall good acceptability of communicating test results by text messaging notification.^3,4,17–21^ For instance, 52% of patients in a US study and 69% of UK patients opted for texting.^7,9^ Most published studies on the practical acceptability of text messaging have been conducted for bacterial STIs (chlamydia and gonorrhea). However, text messaging has also already been used to reduce loss to follow-up after HIV serology.^13^ Finally, a few studies have suggested that patients' theoretical preferences may vary according to patient profile, such as age, gender, sexual orientation or economic status.^4,21,22^ It is now necessary to explore the influence of patients' profile on the practical acceptability of text messaging for both bacterial and viral sexual infections.

The aim of this study was to explore patients' profile associated with nonparticipation in a program of notification through text messaging after STI/HIV testing.

## MATERIALS AND METHODS

### Setting

The study was conducted in a free center for information, screening and diagnosis for HIV and STI (CeGIDD) located in a university hospital in Paris. The center offers screening for HIV, HBV, HCV, syphilis, chlamydia and gonococci. During pre-test counseling, the physician evaluates the patient's risk-taking behavior and prescribes appropriate screening tests for each patient. Patients can remain anonymous or can give their name as they prefer. Whether the patient chooses to remain anonymous for screening or not, he or she is given an anonymity number. This number is used by the physician and nurse to call the patient in the waiting room. The consultation and blood testing take place in a closed room to ensure confidentiality. Since August 2016, patients are offered the possibility of being notified about their test results by SMS, rather than systematically coming to the center. In accordance with medical guidelines, if one of the screening tests performed is positive, results are not directly given in the text message. Patients are invited to return to the center to obtain their results and receive appropriate guidance. Participation in the SMS program is proposed and explained by the receptionist at the center, who gives the patients an information leaflet. During October 2016, 396 patients were screened for STIs including 360 who completed a short anonymous self-administered questionnaire in the waiting room (available in French and in English). Among the 396 screened patients, 271 participated in the SMS program (68%) and 122 (32%) did not.

### Study Population

The study population included all patients who did not participate in the SMS program and who completed the anonymous self-administered questionnaire (n = 100). Although a standardized procedure aims to ensure that the same information is delivered to all patients regarding SMS notification, the time spent by the reception agent may vary depending on the influx of patients. Because this reception context is a potential confounding factor difficult to measure, a chronological matched case-control study design was chosen.^23,24^ Each of the 100 patients of the study population was matched with the next patient coming for STI screening who participated in the SMS program and had completed a questionnaire. The median time between the visits of a pair consisting of one case and one control patient was 30 minutes (Q1-Q3 [10–90]).

### Data

Data on consultations and test results were routinely registered in the center database. This database included date of consultation, anonymity status (whether the patient decided to remain anonymous or not), participation in the SMS program, year of birth, gender, screening tests prescribed by the doctor and performed (HIV, HBV, HCV, syphilis, chlamydia and gonococci), test results, date of face-to-face delivery of results and/or date of SMS according to participation or nonparticipation in the SMS program and to the nature of the results (all negative or at least one positive result). As the various screening tests implied diseases which have very different levels of seriousness, a summarized single binary variable was created as an indicator of the seriousness of the diseases screened: screening for at least HIV, HBV and HCV vs other combinations.

The self-administered questionnaire included:

- sociodemographic data: gender, country of birth, place of residence, work status (employed, in training, no professional activity), educational level, health insurance coverage (full coverage for patients covered both by statutory health insurance and complementary voluntary health insurance, basic coverage for patients covered only by statutory health insurance, state assistance related to low income or illegal immigrant status, or no health insurance),

- data on sexual behavior: sexual orientation, number and type of sex partners (regular and/or occasional),

- data on the STI screening process: person who suggested screening (the patient himself/herself or someone else), previous screening for HIV.

Due to the small number of patients born in Asia and Africa who had an university diploma, country of birth and educational level were combined in a single variable with three categories: *University diploma when born in Europe or America*, *No university diploma when born in Europe or America,* and *Born in Asia or Africa*. Place of residence was used as a proxy for socio-economic status as the high rents in Paris compared with the suburbs lead to marked social stratification.

### Statistical Analysis

Characteristics of patients and of their screening tests were compared according to participation in the SMS program (no-SMS group vs SMS group) using the Stuart-Maxwell test for matched-pair case-control data with multiple discrete levels of the exposure variables.^25,26^ Factors associated with nonparticipation in the texting program were studied using univariate and multivariate conditional logistic regression models. Multivariate analysis included factors selected by a backward stepwise selection method with a 0.2 significance level for removal from the model. Statistical analyses were performed using STATA/SE 13.1 (Stata Corporation, College Station, TX, USA).

## RESULTS

The infections screened are shown according to participation in the SMS program in Table 1. Almost all patients were screened for HIV and more than half of patients were screened for other STIs. Patients were screened for a median of 4 infections (Q1-Q3 [3–5]).

**TABLE 1:**
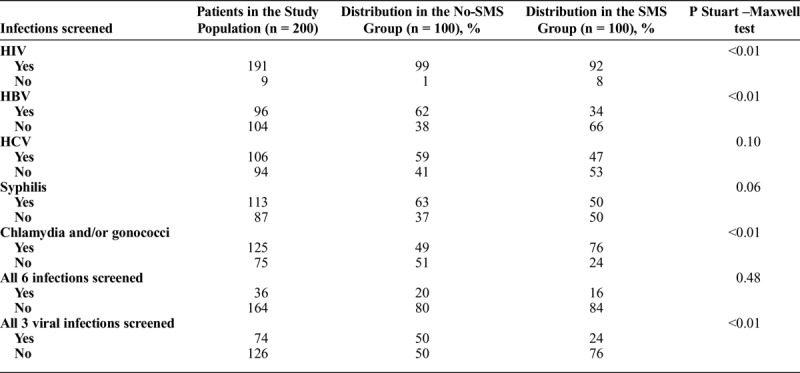
Infections Screened according to Participation in the SMS Program among the Matched Case-Control Study Population (n = 200)

The no-SMS group was compared with the SMS group (Table 2). In our study population, 9.5% of patients had at least one positive result after STI screening. This proportion was similar in both groups (*P* = 0.80). However, patients from the no-SMS group were more often screened for more serious STIs (at least HIV, HBV and HCV) than patients from the SMS group (50% vs 24%, *P* < 0.01). Except for gender, all other socio-demographic factors studied were associated with nonparticipation (Table 2): age, social background, place of residence, work status and health insurance coverage. Patients from the no-SMS group were less likely to respond to questions on their sexual orientation and sexual partner(s).

**TABLE 2:**
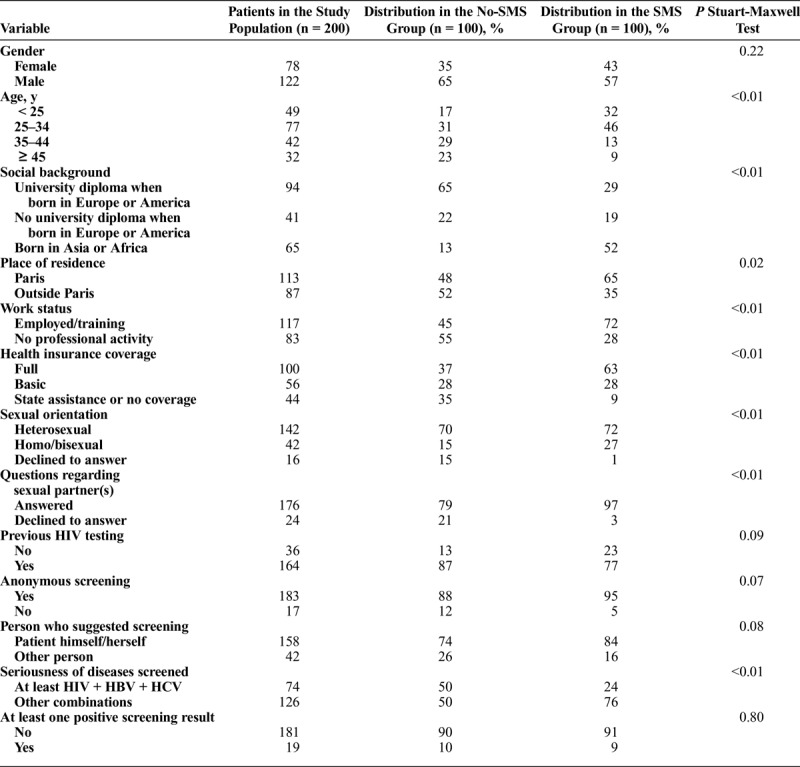
Characteristics of Patients and Screening Tests According to Participation in the SMS Program among the Matched Case-Control Study Population (n = 200)

Results of univariate and multivariate conditional logistic regressions for nonparticipation in the SMS program (i.e. belonging to the no-SMS group) are presented in Table 3. The backward stepwise selection method retained seven variables in the multivariate model. In multivariate analysis, nonparticipation was higher among patients aged 35 years and over, those with a less favorable social background and those living outside Paris. Sexual orientation and non-response to questions regarding sexual partner(s) were associated with nonparticipation in the SMS program. Lastly, patients who had previously been tested for HIV were also less likely to participate in the SMS program.

**TABLE 3:**
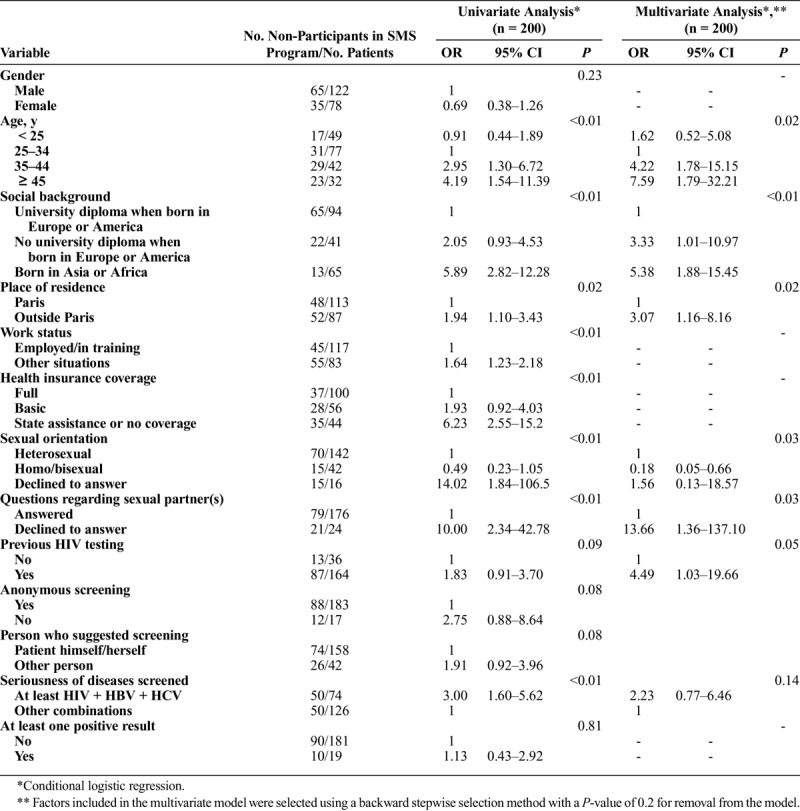
Characteristics Associated with Nonparticipation in the SMS Program Among the Matched Case-Control Study Population (n = 200)

## DISCUSSION

In a free screening center in Paris, 68% (95% CI [64–73]) of patients screened for STIs or HIV agreed to participate in the SMS program after testing. This acceptance rate was similar to the 69% acceptance rate observed in a UK study conducted in a genitourinary clinic^9^ and higher than that observed in the United States (52%).^7^ In our matched case-control study, we found that participation in the SMS program was not related to screening results, as the proportion of positive results did not differ between the no-SMS group and the SMS group. In multivariate analysis, the seriousness of the disease screened (at least HIV and viral hepatitis) was also not associated with SMS program participation. Participation was not related to STI screening characteristics and appeared to be related only to patient characteristics, in particular sociodemographic characteristics and sexual behavior.

The four characteristics describing the social profile of the patient were significantly associated with nonparticipation in the SMS program in univariate analysis (social background, place of residence, work status, health insurance coverage). Consistently, on all four variables, less favorable social conditions were more frequent in the no-SMS group than in the SMS group. The stepwise procedure led us to include only social background and place of residence (living outside Paris being a strong indicator of socioeconomic status due to the lower housing rental costs). In this multivariate model, nonparticipation in the SMS program was higher among patients born in Asia or Africa. In the United States, non-white patients were less likely to participate in an SMS program.^7^ In our study, patients with no university diploma also had a higher probability of nonparticipation in the SMS program. Socially less favored patients could be less comfortable with SMS because it is a written form of communication that they find more difficult to deal with than oral communication. Similarly, a study conducted among women attending an STI clinic in the United States showed that patients with a lower socioeconomic status were less likely to use text messaging.^27^ However, a few studies exploring patient preferences, rather than actual participation, concluded that socially less favored patients tended to indicate greater preference for text messaging for receiving STI results than socially more favored patients.^4,19^ The origin of this discrepancy between preferences and actual behavior concerning text messaging among socially less favored patients needs to be investigated.

Nonparticipation in the SMS program also seemed linked to sexual behavior. Patients declaring homosexual or bisexual relations were more likely to participate in the SMS program than patients declaring heterosexual relations. Moreover, patients who did not respond to questions regarding their sexual partner(s) were less likely to participate than patients who responded to these questions. Studies have shown that refusal to participate in sexual behavior research is associated with a less open attitude toward sex and with feelings of guilt and shame about sex.^28^ Based on our results, it can be postulated that patients who are less comfortable with (their) sexuality could be less willing to receive their STI results by SMS and could prefer face-to-face feedback. The lower level of acceptability of SMS among these patients could reflect concerns regarding confidentiality. This would also explain their declining to answer questions on sexual partner(s). In the literature, privacy concerns appear to be a recurring barrier to participation in text messaging programs, with the fear that another person might read the message.^17,22,29,30^

In our study, participation in the SMS program did not differ according to the patient's gender, but did differ according to age. Older patients had a significantly higher probability of nonparticipation, with a mean age of 29 years in the SMS group and 36 years in the no-SMS group. Among patients in the United States, texters were also younger than non-texters.^7^ Following the same logic as that discussed above for sexual behavior characteristics, this age effect could reflect a feeling of greater unease among older patients during the STI screening process because they may tend to consider that, in view of their age and prevailing social norms, they should be engaged in a stable relationship that does not require STI screening. Young people may feel more comfortable in discussing sexuality and reporting information regarding sexual behavior.^28,31,32^ Moreover, the relation that we observed between nonparticipation in the SMS program and age may be confused by unmeasured factors, such as marital status (this was not available in our study). Patients older than 35 years may be more likely to be married than younger ones. Therefore, this higher nonparticipation among older patients could be related to a higher proportion of persons involved in extramarital relationships and more worried about privacy. Feeling less comfortable with STI screening and privacy concerns may both be factors that lead patients to choose to receive their results face-to-face from a doctor rather than to be notified by SMS. Lastly, although our population was relatively young (90% of patients included in the study were younger than 47 years), less ease with use of mobile technology among the older patients linked to a generation effect cannot be excluded, as text message use is associated with younger age.^27^

Three limitations could have affected our results. Firstly, the short self-administered questionnaire was available only in French and in English. This was a possible barrier for non-French patients who could have been excluded from the study population because they were unable to complete the questionnaire. Secondly, to keep the questionnaire short and easy to complete, only a proxy indicator (place of residence) measured the standard of living. In the Paris region where there is a marked difference in rents between the city and its suburbs, place of residence is a very good indicator of standard of living that can reliably be collected in a short self-questionnaire. Complementary data on household income would have been useful, but a much longer questionnaire would have been needed for it to be efficiently collected. Lastly, the size of the study population did not allow specific analysis of small subpopulations. For example, it was not possible to explore as a single analytical category patients born in Asia or Africa who had a university diploma, or the groups of men who have sex with men (MSM) or women who have sex with women (WSW).

In conclusion, the global acceptability of this text messaging program after HIV and STI screening appeared quite good, with 68% of patients screened agreeing to participate. However, patient profile was an important factor in participation in this SMS program. Nonparticipation was higher among patients living in less favorable social conditions, patients older than 34 years and patients who did not respond to questions regarding sexual behavior. These factors may reflect a preference for oral communication and/or less familiarity with use of such technology in a health context. Patients may not be comfortable with the STI screening process and may have privacy concerns. Text messaging is generally acceptable and efficient for the transmission of negative results, saving time for both healthcare professionals and patients. However, participation in such a program varies according to patient profile. Moreover, SMS notification is not suitable for patients who have difficulty in reading. Consequently, several options should be proposed for delivering the results of STI screening to increase the probability of patients being informed of their STI test results.
